# Antipsychotics during transition from adolescence to adulthood

**DOI:** 10.1192/j.eurpsy.2026.10389

**Published:** 2026-07-31

**Authors:** P. Mohr

**Affiliations:** 1 National Institute of Mental Health, Klecany; 2 Third Faculty of Medicine, Charles University, Prague, Czech Republic

## Abstract

**Abstract:**

The period of transition from adolescence to adulthood is critical for continuity of psychiatric care. Discontinuity of treatment, loss of follow-up, can severely affect outcome of psychiatric conditions. As indicated by a survey among European mental health professionals, fragmented delivery and disruption of care is perceived as one of the barriers in access to mental health services. Currently, we are facing big surge of mental health problem among children and adolescents. This is also evidenced by the sharp increase in consumption of psychotropic drugs. The number of young people (under age of 17 years) in the Czech Republic, who were prescribed antipsychotics, went up between 2015 and 2025 by 25%. The same pattern can be observed in other countries, as well. Antipsychotic treatment of adolescents requires close scrutiny, especially in those who are at risk of transition to psychosis. The data indicate that antipsychotics may not be more effective in psychosis prevention than other interventions, both pharmacological and non-pharmacological. Moreover, antipsychotics, typically off-label, have much broader variety of indications in children adolescents than just psychotic disorders (e.g., mood disorders, disruptive behavioral disorders, autism spectrum disorders, etc.). Although the spectrum of side effects induced by antipsychotics in youngsters and adults is similar, young patients may be more sensitive, especially to metabolic and endocrine adverse events. Another controversial issue is the use of long-acting formulas in this population. Antipsychotic treatment between adolescence and adulthood thus requires both continuity and careful deliberation of right indication, as well as taking into consideration its different tolerability and safety.

**Disclosure of Interest:**

None Declared

